# Chemical profiling of *Egregia menziesii* and anti-inflammatory potential in LPS-activated RAW 264.7 macrophages

**DOI:** 10.1007/s11033-026-12685-y

**Published:** 2026-09-05

**Authors:** Omar Arroyo-Xochihua, Alberto Sánchez-Medina, Óscar López-Franco, Tatiana N. Olivares-Bañuelos, Rossana C. Zepeda

**Affiliations:** 1 Doctorado en Ciencias Biomédicas, Centro de Investigaciones Biomédicas, Universidad Veracruzana, Xalapa, Veracruz México; 2 Laboratorio de Farmacología y Quimiometría, Instituto de Química Aplicada, Universidad Veracruzana, Xalapa, México; 3 Laboratorio de Medicina Traslacional, Universidad Veracruzana, Xalapa, Veracruz México; 4 Instituto de Investigaciones Oceanológicas, Universidad Autónoma de Baja California, Ensenada, Baja California México; 5 Laboratorio de Biomedicina Integral y Salud, Centro de Investigaciones Biomédicas, Universidad Veracruzana, Xalapa, Veracruz México; 6 Laboratorio de Diagnóstico Molecular, Centro de Investigaciones Biomédicas, Universidad Veracruzana, Xalapa, Veracruz México

**Keywords:** Immune system, Seaweeds, Cytokines, Inflammation, Chemometrics

## Abstract

**Background:**

*Egregia menziesii* (*E. menziesii*) is recognized for its anticancer and hypoglycemic properties. Chemically, it contains compounds with potential anti-inflammatory activity. Within the immune system, macrophages play an essential role in containing and eradicating pathogens through phenotypic polarization.

**Methods and results:**

The ethanol and water (1:1 v/v) (EtOH: H_2_O), extract of *E. menziesii* was obtained and fractionated. The chemical profiles of the extracts featured signals linked to sulfated polysaccharides and mannitol. Cell viability was evaluated at various concentrations with MTT assay and confirmed the safety (≥ 90% of cell viability) of the extract and fractions for cellular experimentation. The scratching assay demonstrates the impact of the EtOH: H_2_O and aqueous fraction (FR_Aq) treatments, evidenced by a reduction of up to 40% and 10% in the free scratched area at 24 and 48 h, respectively. The phagocytosis assay, determined by the neutral red assay, shows that EtOH: H_2_O treatment decreases the process to levels below those of the LPS- control group (< 120%). Proton signals of the extract and fractions were correlated with the scratched area value via Multivariate Data Analysis (MVDA) of the ^1^H-NMR data of the extract and fractions and the results from scratching assay showed the signals at *δ* 0.6–3 ppm as the main contributors to the activity. Additionally, interleukin-1β (IL-1β) and interleukin-6 (IL-6) expression levels were determined by RT-qPCR and ELISA, showing that the EtOH: H_2_O extract could influence their mRNA expression (Fold change < 0.5 and < 3, respectively) and protein levels.

**Conclusions:**

*E. menziesii* safely regulates macrophage behavior, IL-1β and IL-6 release and promotes scratch area reduction. The immunomodulatory effect could be linked to the presence of sulfate polysaccharides and mannitol.

**Graphical abstract:**

**Figure:**
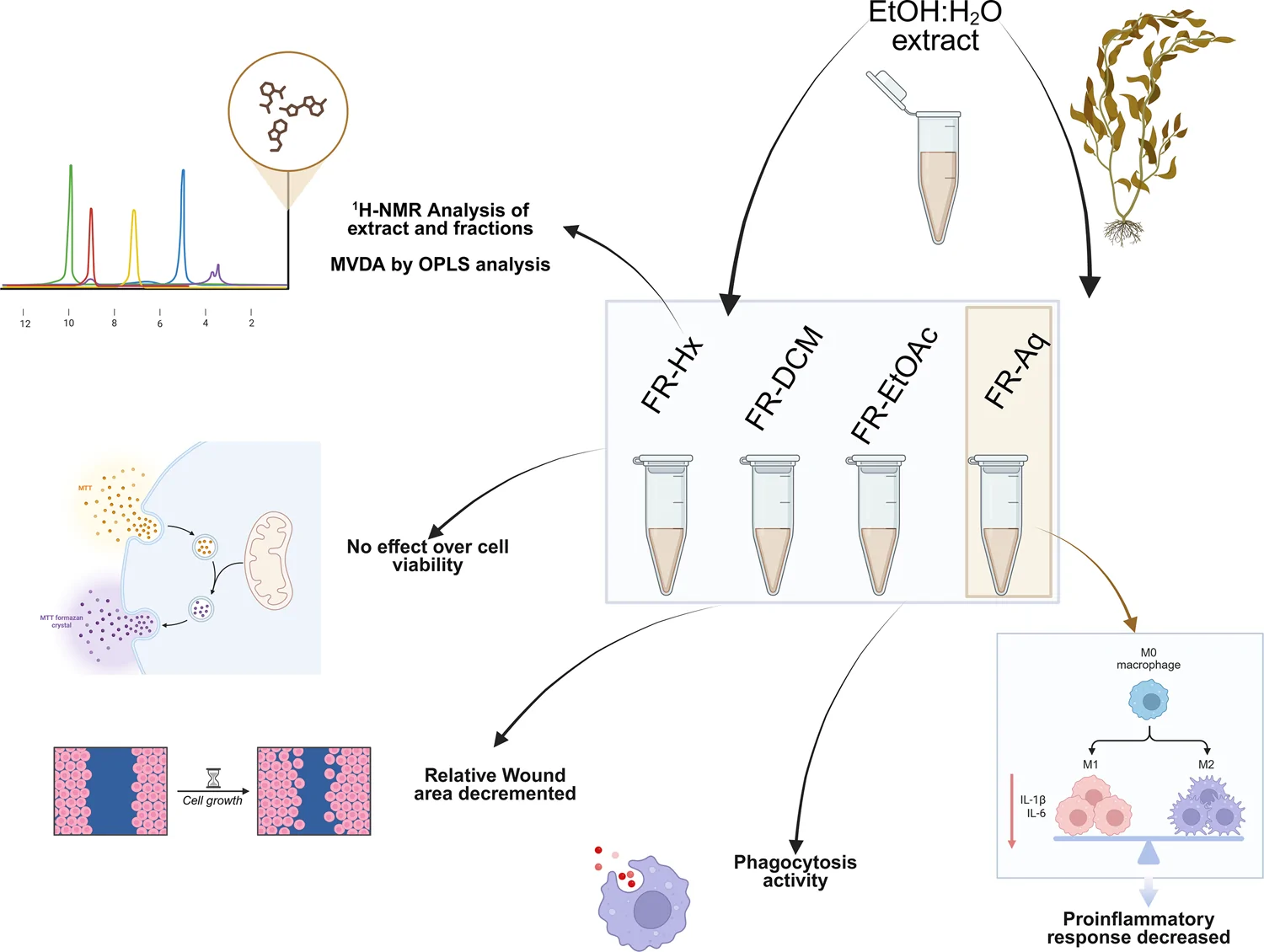

**Supplementary Information:**

The online version contains supplementary material available at https://doi.org/10.1007/s11033-026-12685-y.

## Introduction

Inflammation is a process regulated by the immune response, involving interactions among cellular, physical, and chemical mechanisms in response to external antigens or those produced endogenously. Although several anti-inflammatory and immunomodulatory drugs are available, providing numerous therapeutic advantages, the widespread use of non-steroidal anti-inflammatory drugs (NSAIDs) is linked to adverse effects [1]. Moreover, multiple pharmacoepidemiological studies have shown an excessive increase in NSAIDs use in recent years, which may contribute to the rising incidence of toxicity [2]. Herbal remedies are frequently used alongside allopathic medications as adjuvants [3]. The functional metabolites found in medicinal plants have served as a basis for treating numerous health conditions and continue to be a promising source for research and development of new drugs [4]. To date, numerous functional metabolites with diverse chemical structures and pharmacological properties have been identified in plants from both terrestrial and marine ecosystems. Between 1981 and 2019, over 50 natural products and their derivatives for the treatment of inflammation were approved. However, studies on the potential pharmacological effects of marine organisms represent only a small fraction [5, 6].

Macroalgae occupy the primary trophic level within the marine ecosystem and, together with microalgae, play a vital role in maintaining the equilibrium of abiotic and biotic factors that influence marine life. Some seaweed species have been used in food and medicinal applications across various regions worldwide, with India, China, and Japan leading in production in Asia; however, Mexico, Chile, and Canada are also significant contributors to macroalgae production in the Americas [7]. Mexico’s extensive coastline supports a diverse range of marine macroalgae, including numerous endemic species. This offers a unique opportunity to explore their industrial and biomedical applications [8].

Macroalgae are classified into three main groups: *Rhodophyta* (red), *Chlorophyta (*green), and *Phaeophyta (*brown) [9]. Seaweeds are recognized for their rich levels of polysaccharides, vitamins, and minerals. Recently, bioactive substances such as polyphenols, peptides, lipids, and pigments have attracted increasing interest due to their diverse biological activities [10]. The *Lessoniaceae* family comprises brown seaweeds that synthesize compounds such as polysaccharides, alginic acids, laminarins, fucoidans, phlorotannins, and diterpenes [11, 12], which have been extensively demonstrated to possess anti-inflammatory properties [13–16].

*E. menziesii* is a perennial brown algae classified within the phylum *Ochrophyta*, the family *Lessoniaceae*, and the genus *Egregia*. It is native to the west coast of North America, with a geographical range extending from Alaska to Baja California, Mexico, making it an algae with a limited distribution. Taxonomically, it is closely related to *Ecklonia cava*, *Eisenia arborea*, and *Undaria pinnatifida*, as well as algae belonging to *Alariaceae*, *Aaraceae*, and *Arthrotamnaceae*. Chemically, it contains a considerable amount of proteins, ash, phlorotannins (phloretol, fucophloretol, and phloroglucinol), and compounds containing sulfoxide and sulfate groups. It also shows low lipid content and DPPH-targeted radical scavenging effects [17]. *E. menziesii* extracts are considered safe for administration, as evidenced by the use of its methanolic extract in *Artemia salina*, nervous system cell lines, and human peripheral blood lymphocytes [18]. However, its effect on the immune system has not yet been elucidated. The anti-inflammatory effects of seaweeds are commonly assessed by examining the production of pro-inflammatory cytokines and the expression of various genes associated with inflammatory signaling pathways within immunological processes [19]. In this system, macrophages are part of the first line of defense and are responsible for numerous processes in both innate and adaptive immunity. Particularly, RAW 264.7 macrophages are a classical cell line used to measure inflammatory parameters [20, 21]. Therefore, in this study, RAW 264.7 macrophages are used to assess the effects of *E. menziesii* EtOH: H_2_O extract and its fractions on inflammatory processes. Additionally, the chemical profiling of *E. menziesii* extract and its fractions was carried out using ^1^H-NMR. Finally, multivariate data analysis (MVDA) by Orthogonal Partial Least-Squares (OPLS) allowed the characterization of the type of functional metabolites associated with the anti-inflammatory effects.

## Materials and methods

### Chemicals and reagents

Thiazolyl Blue Tetrazolium Bromide (MTT) (M2128-500MG), Neutral red (N7005-25G), Dimethyl sulfoxide-*d6* (DMSO-*d*_*6*_) (296147-25G), 4-Hydroxybenzoic acid (H20059-100G), curcumin (C1386-10G), and LPS (Escherichia coli 0111:B4) were obtained from Sigma. Indomethacin (57413) was obtained from Fluka. QIAzol lysis reagent (79306) was obtained from Qiagen, High-Capacity RNA-to-cDNA TM Kit (Cat. 18080-093), and SYBR Green PCR Master Mix (4309155) were from Applied Biosystems. The IL-1β ELISA kit (ab197742) and IL-6 ELISA kit (ab100713) were obtained from Abcam. The cell culture medium, Roswell Park Memorial Institute (RPMI) 1640 Medium (L0500), the penicillin-streptomycin antibiotic solution (BIO-L0022-100), and fetal bovine serum (FBS) (S1560-500) were acquired from Biowest.

### Extract preparation and fractionation

A total of 25 g of *E. menziesii* collected from “Campo No. 5”, Punta Banda, Baja California, Mexico (31°44′3.45″ N; 116°43′39.95″ W) during 2014 were used in this study. Dry fronds were ground into a fine powder, then 225 mL of a solvent mixture of EtOH: H_2_O (1:1 v/v) was added. This mixture was subjected to ultrasonic treatment for 60 min at 25 °C and 25 kHz. Subsequently, the mixture was filtered under vacuum through a Whatman Grade 5 filter paper. Solvents were eliminated under reduced pressure using a Rotary evaporator R300 (Büchi, Flawil, Switzerland). The crude extract was transferred to a pre-weighed vial and stored at 4 °C, protected from light. The crude extract was suspended in methanol: water (5:95) (1:25 w/v) and subjected to fractionation using solvents of increasing polarity: hexane, dichloromethane, and ethyl acetate with 4 washes. The residual aqueous fraction was also recovered. The recovered fractions were dried under reduced pressure using the Rotavapor R300 and stored in amber glass bottles at 4 °C.

### ^1^H-NMR seaweed extract and fractions profile

Ten mg of EtOH: H_2_O extract of *E. menziesii* and each fraction were dissolved in 600 µL of Dimethylsulfoxide-d6 (DMSO-*d6*) with tetramethylsilane (TMS), and 30 µL of 4-HBA (4-hydroxybenzoic acid at 10 µg/mL); then, transferred to an NMR tube. Spectra were recorded in duplicate using an Agilent Unity INOVA 500 MHz spectrometer at 25 °C. Water peak was suppressed using a pre-saturation method, and the measurement parameters were as follows: number of data points, 32 K; spectral bandwidth, 8012 Hz; acquisition time, 2.045 s; 128 scans. Spectra were processed using MestReNova v14.2.0 software (Mestrelab Research S. L.). The processing consisted of baseline correction, phase correction, and referencing of the spectra to the TMS signal (used as an internal reference) at 0 ppm. The 4-HBA fifth-proton signal was also used to normalize all spectra to 100. Proton identification for compound characterization was conducted by comparing the δ (ppm) values of the emulated spectra, reference compounds, and the Human Metabolome Database (HMDB).

### Preparation of control, seaweed extract, and fractions for in vitro experiments

For subsequent use in cell culture, 1 mg of the extract or fraction of *E. menziesii* was reconstituted in 100 µL of DMSO and subsequently, culture medium was added to 1 mg/mL. The extract was filtered using a sterile hydrophilic filter with a 0.22 μm pore size and a 33 mm diameter, performed in a laminar-flow hood. Control curcumin (CUR) and indomethacin (INDO) were prepared in the same way.

### Macrophages RAW 264.7 cell culture

The murine macrophage RAW 264.7 cell line (ATCC-TIB-71) was cultured as a monolayer in various T25 flasks. Cells were maintained in RPMI medium supplemented with phenol red, L-glutamine, 10% uncomplemented Fetal Bovine Serum (FBS), and 1% antibiotic solution, under conditions of 5% CO_2_ at 37 °C. Cells were passaged up to ten times and maintained at approximately 90% confluency. The initial cell seeding density was determined by counting cells in a Neubauer chamber using the trypan blue exclusion method.

### Cell viability

RAW macrophages were cultured at a density of 1 × 10^4^ cells/well in 96-well plates with supplemented RPMI medium. After the cells reached confluence and the starvation period, the macrophages were treated with 0, 12.5, 25, 50, 100, and 1000 µg/mL of the prepared extract. The 1000 µg/mL fraction was discarded. The incubation periods were 24 and 48 h. As an antiproliferative control, cells were treated with hydrogen peroxide (H_2_O_2_) at 2 mM in the culture medium. A vehicle without extract or fraction extract was utilized. Following treatment and incubation, and after washing with Phosphate Buffered Saline (1X PBS), mitochondrial reduction was assessed using the MTT assay, in which 2 mg/mL MTT was prepared and added to supplemented RPMI medium (100 µL/well). The samples were incubated for 2 h at 37 °C. The medium was then aspirated, and the formazan crystals were dissolved in 100 µL of DMSO. Absorbance was measured at 570 nm using a microplate spectrophotometer (Agilent BioTek Epoch Microplate Spectrophotometer, Agilent Technologies, Santa Clara, CA, USA). The formazan percentage correlates with the number of viable and active cells (%). Data were expressed as the relative percentages of viable cells.

### Scratch assay

RAW 264.7 cells (1 × 10^6^ cells/well) were seeded in a 6-well culture plate and incubated for 24 h to achieve approximately 80% confluency. The cells were then subjected to serum starvation for an additional 24 h. Following this starvation period, scratched area were generated using a 200 µL micropipette tip. Macrophages were subsequently treated with 100 µg/ml of *E. menziesii* extract or its fractions, in conjunction with pharmacological and natural control agents. LPS was administered one hour after treatment to 2.5 µg/ml as final concentration. The incubation periods extended to 24 and 48 h. Post-incubation, the cells were washed with 1X PBS. Photographs of the scratched area were captured, and were quantified using ImageJ software version 1.54r [22]. The extent of closing was estimated by calculating the recovery percentage using the following formula: R (%) = [1 - (Scratched area at Tt/Scratched area at zero time (T0))] × 100, where T0 denotes the initial scratched area at zero hours and Tt the total time.

### Assessment of phagocytic activity

To measure phagocytosis, cells were seeded at a confluence of 1 × 10^4^ cells/well in 96-well plates with supplemented RPMI medium for 24 h. Following a 24 h starvation period, macrophages were treated with 100 µg/ml of *E. menziesii* extract or fractions, along with pharmacological and natural controls. LPS was administered after a one-hour treatment period to 2.5 µg/ml as final concentration. The incubation period lasted 24 h. Following treatment and incubation, the cells were rinsed with 1X PBS, and 100 µL of 0.0075% neutral red was added to each well. After a 2-hour incubation, the cells were washed twice with 1X PBS to eliminate any remaining red dye. Cells were disrupted using an acetic acid: ethanol (1:1) solution. Finally, absorbance was measured at 570 nm with a microplate reader (Agilent BioTek Epoch Microplate Spectrophotometer, Agilent Technologies, Santa Clara, CA, USA). The percentage correlates with the number of active viable cells (%). Data were expressed as the relative percentages of active viable cells.

### Total RNA isolation and reverse transcription quantitative polymerase chain reaction PCR (RT-qPCR)

RAW 264.7 cells (1 × 10^6^ cells/well) were seeded in a 6-well culture plate and incubated for 24 h to attain approximately 80% confluence. Subsequently, the cells were subjected to serum starvation for an additional 24 h. Following the starvation period, macrophages were treated with 100 µg/mL of *E. menziesii* extract or fraction, alongside pharmacological and natural controls. LPS was administered one hour after treatment to 2.5 µg/ml as final concentration. The incubation period lasted for 24 h. Total RNA was isolated using QIAzol lysis reagent. The RNA quality and integrity were assessed via agarose gel electrophoresis and a Thermo Scientific Multiskan SkyHigh Microplate Spectrophotometer equipped with a µDrop Duo plate. Real-time PCR was performed to assess the mRNA expression levels of IL-1β, IL-6, and GAPDH. The primer sequences are listed in Online Resource 1 [23–25]. GAPDH served as an endogenous control. Quantitative PCR was performed using a Thermo Scientific PikoReal 96-well plate qPCR system. The relative gene expression levels were quantified using the 2 − ΔΔCT method. The data are presented as fold changes in target gene expression normalized to the internal endogenous control gene.

### Measurement of IL-6 and IL-1β

The expression levels of IL-6 and IL-1β in macrophage culture medium and extracts were quantified utilizing commercial ELISA kits. The cells underwent pretreatment for 24 h. Cytokine expression in cells was assessed according to the manufacturer’s protocol.

### Data analysis

Data are expressed as the mean ± standard error of the mean (SEM). In accordance with the normality and homoscedasticity tests, data were analyzed using one- or two-way ANOVA, followed by *post hoc* Dunnett, Sidák, or Tukey tests, as appropriate. GraphPad Prism^®^ version 10.5.0 was used for all statistical analyses. Differences between groups were considered statistically significant if *p* < 0.05. For MVDA, OPLS analysis was conducted using SARTORIUS SIMCA (Sartorius Stedim Data Analytics AB, version 18.01), with Pareto scaling applied. Proton NMR chemical shifts (spectral signals) served as X-variables, while the decrement in Scratched area (%) was treated as the Y-variable. Model validation employed R^2^X, R^2^Y, and Q^2^ values.

## Results

### ^1^H-RMN profiling of *Egregia menziesii*

Extraction using a solvent mixture of ethanol and water (v/v) has been demonstrated to improve the obtention of various compounds. The yield from the EtOH: H_2_O extract confirms that this method extracts a substantial number of components. Subsequent fractionation indicates that the dichloromethane and ethyl acetate fractions (FR-DCM and FR-EtOAc, respectively) exhibit the lowest yields, while the FR-Aq demonstrates the highest (Online Resource 1).

**Fig. 1:**
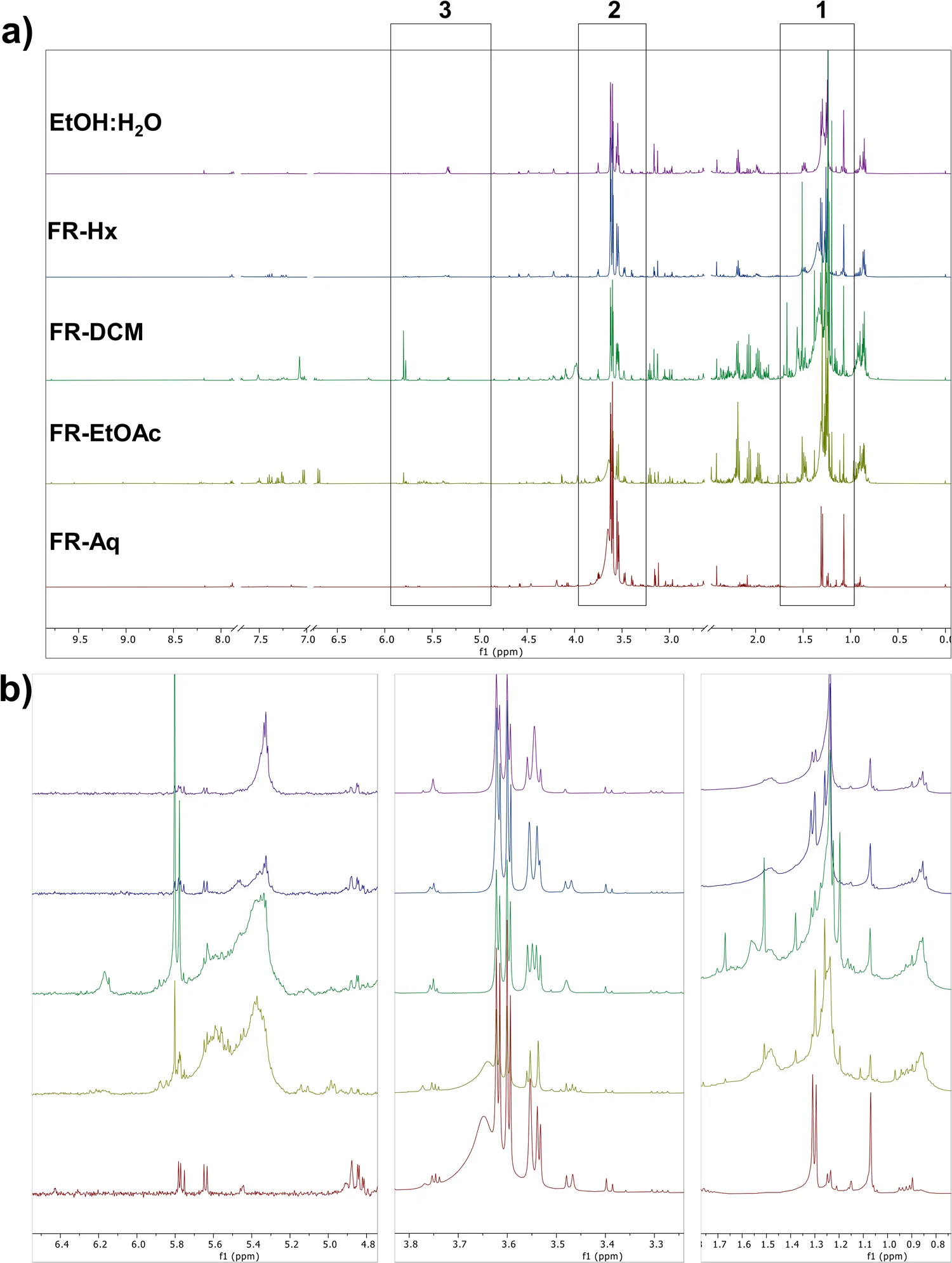
The fingerprint of *E. menziesii* EtOH: H_2_O extract and its fractions reveals protons associated with anti-inflammatory compounds. a) Fingerprint the crude extract and fractions. Chemical shifts are expressed in parts per million (ppm). The reference used was TMS, and the internal standard for normalization was 4-HBA. b) The compounds of interest are indicated in a rectangle and numbered as follows: (1) Fucoidan methyl group, at δ 1.10–1.25 ppm; (2) Mannitol at δ 3.0-4.5 ppm; (3) Anomeric proton regions of fucoidan (H1α and H1β) at δ 5.0-5.8 ppm

The solvents of increasing polarity effectively isolate distinct protons, as demonstrated by one-dimensional ^1^H-NMR spectra (Fig. 1). The chemical shifts along the x-axis represent different proton compounds, whereas the signal intensities along the y-axis indicate the concentration of the respective chemical constituents. A diverse array of proton signals within the ranges of alkyl-CH, -OH, -NH, alkenes, and aromatic compounds is observed. It is noteworthy that the signals at *δ* 1.10–1.25 ppm, 5.0-5.8 ppm, and 3–5 ppm can be attributed to sulfated polysaccharides, anomeric fractions of fucoidan, and mannitol, respectively.

### Cell viability effect of *Egregia menziesii* in RAW 264.7 macrophages

**Fig. 2:**
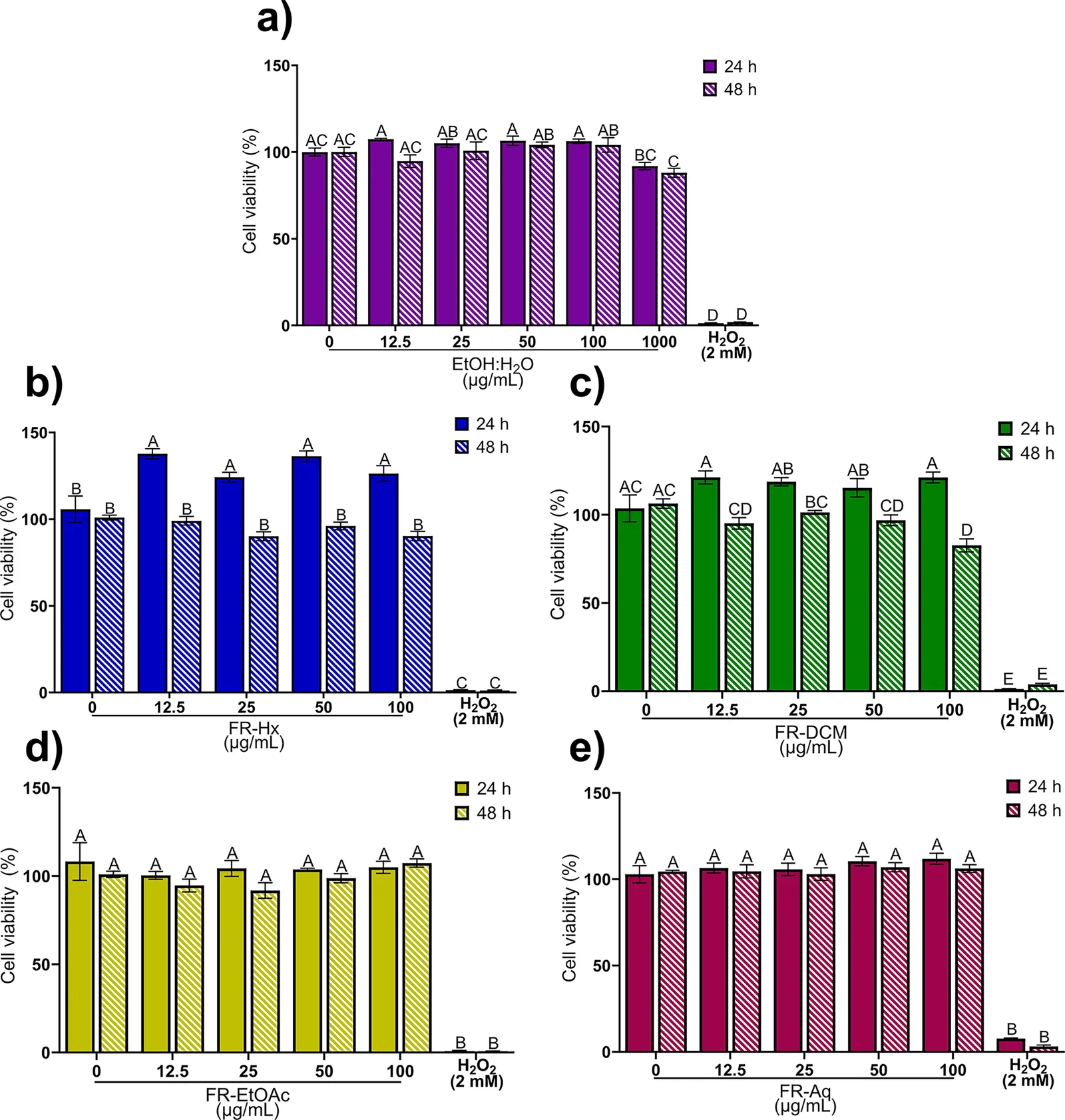
*E. menziesii* exhibits a non-significant effect on cell viability. a) EtOH: H_2_O extract treatments with comparative analysis across different incubation times and concentrations. b-e) Fraction treatments with comparative analysis across various incubation times and concentrations. Data are expressed as mean ± SEM (*n* = 3, triplicate measurements). Solid and striped bars correspond to the seaweed treatments at 24 and 48 h, respectively. Two-way ANOVA was employed, with differences between groups assessed using Tukey’s post hoc test for multiple comparisons. The notation A- D (a) and A- E (b-e): Identical uppercase letters within each bar indicate no statistically significant differences between treatment groups; conversely, different letters denote statistically significant differences within treatments (*p* < 0.01)

The results of the MTT assay indicated a non-significant reduction in cell viability following treatment with *E. menziesii* EtOH: H_2_O extract and fractions, at concentrations up to 100 µg/mL at 24 h, and a non-significant reduction at 48 h, except at 1000 µg/mL for EtOH: H_2_O extract (Fig. 2). According to the regression equation, the half-maximal inhibitory concentration (IC_50_) of *E. menziesii* extract and its fractions, exceeded 1,000 µg/mL at both 24 and 48 h. These findings underscore the safety of administering the EtOH: H_2_O extract and its derived fractions. Two mM H_2_O_2_ was used as a positive control for cytotoxicity, leading to significant macrophage cell death at 24 and 48 h.

### Scratch closure response induced by the EtOH: H_2_O extract of *Egregia menziesii* and its fractions

**Fig. 3:**
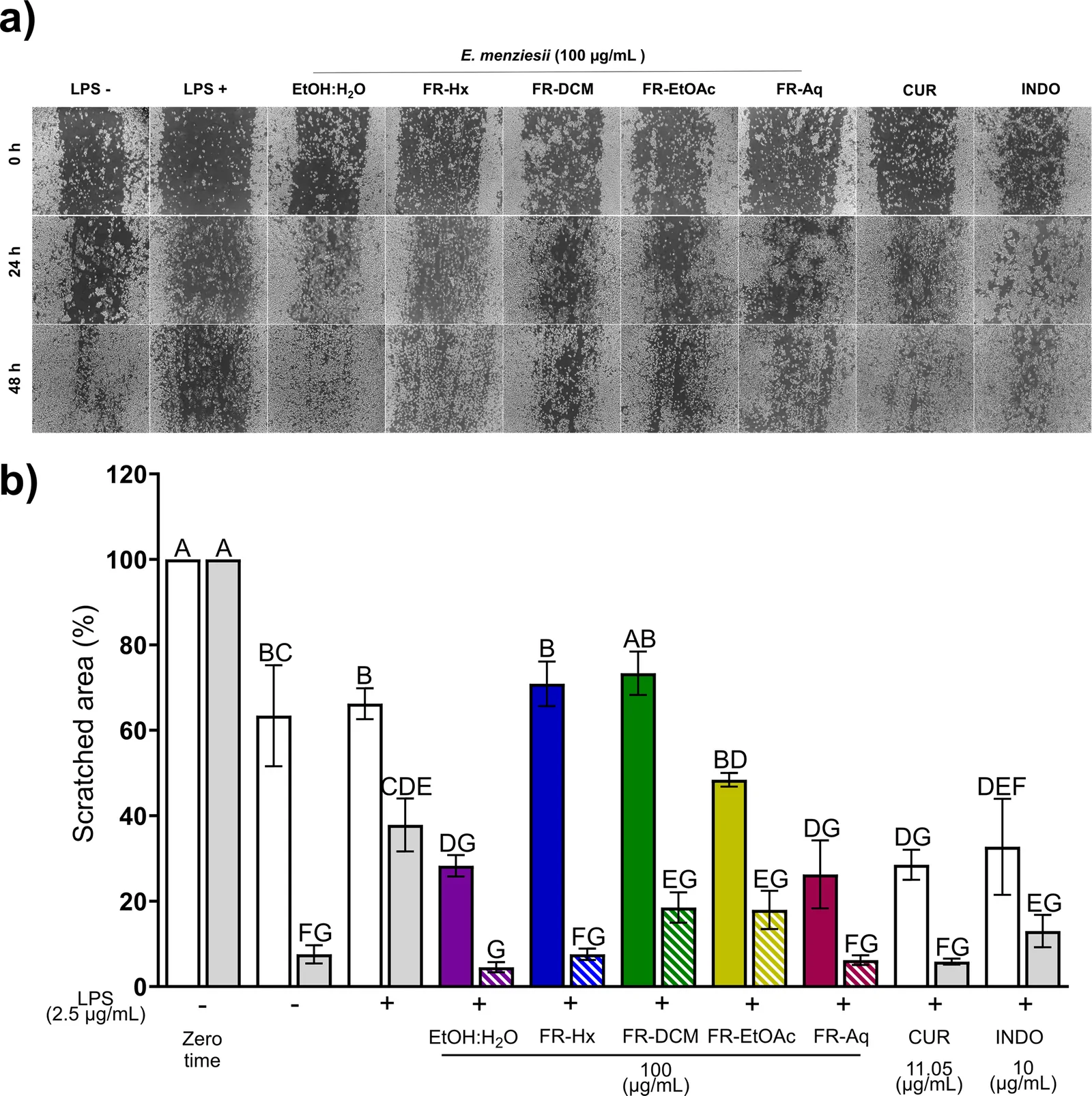
Significant differences were observed compared with the zero-time point in the scratched area. (a) Representative photomicrographs of each treatment (Microscope magnification at 10x). (b) Scratched area quantification. Data are presented as mean ± SEM (*n* = 1, triplicate) at 0, 24, and 48 h. A two-way ANOVA was conducted, and differences between groups were analyzed using Tukey’s multiple-comparisons test. Nomenclature A- G: The same uppercase letters within each bar indicate no significant differences between treatment values; conversely, different letters denote significant differences within treatments (*p* < 0,05). White and grey bars represent control treatments at 24 and 48 h, respectively. Solid and striped bars correspond to the seaweed treatments at 24 and 48 h, respectively

The cell viability assay indicates that the treatments sustain viability close to 100%, consequently, a scratch assay was performed to evaluate whether the extracts and their fractions could facilitate scratch repair and cell migration. Following the experimental procedures, it was observed that the employment of LPS+ had a detrimental effect compared to the LPS– control, as evidenced by insufficient migration at 48 h (*p* < 0.015). Conversely, the EtOH: H_2_O extract significantly reduced the area (*p* < 0.0007 at 24 h, *p* < 0.004 at 48 h) compared to the LPS+ control. Results obtained with FR_Hx, FR_DCM, and FR_EtOAc showed no significant reduction in the area at 24 h. Whereas at 48 h, only the FR_Hx decreases the scratched area (*p* < 0.015). Treatments with CUR (*p* < 0.0007) and INDO (*p* < 0.004) resulted in a significant reduction in the area up to 24 h; however, at 48 h, only CUR continued to significantly decrease the area (*p* < 0.007). The FR-Aq was identified as the most effective option, producing significant differences compared to the LPS+ control group (*p* < 0.003 at 24 h; *p* < 0.0090 at 48 h) (Fig. 3).

### Chemometric analysis of the scratched area closure by the effect of *Egregia menziesii*’s EtOH: H_2_O extract and its fractions

**Fig. 4:**
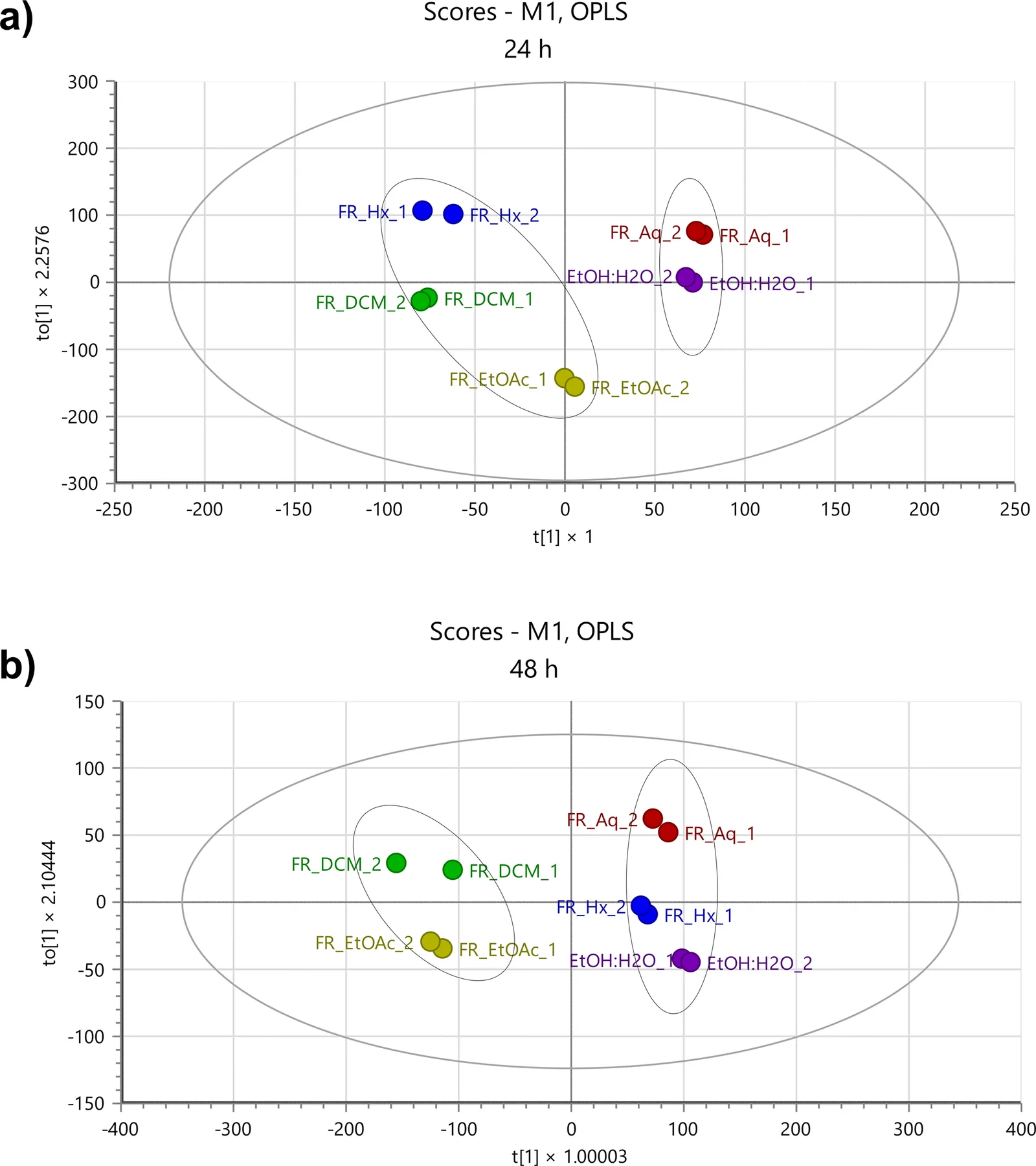
Clustering of extract (EtOH: H_2_O) and fractions of *E. menziesii* with low dispersion by OPLS analysis. (a) 24 h and (b) 48 h. The ellipse interval indicates a confidence interval of 95% by Hotelling´s T2

Figure 4 shows the Score Plot of the OPLS by the clustering of the EtOH: H_2_O extract and fractions of *E. menziesii*. The scratched area value was used as the Y variable to elucidate the contribution of peaks of proton signals in the extract or fractions to the positive or negative impact over scratched area closing. An OPLS model was constructed to obtain the predictive variation associated to the treatment. The score plot showed a clear and robust separation groups marked with circles, along the first predictive component (t [1]) between the EtOH: H_2_O and FR_Aq groups with the FR_Hx, FR_DCM and FR_EtOAc group to 24 h, and between EtOH: H_2_O, FR_Aq and FR_Hx with the FR_DCM and FR_EtOAc group to 48 h (Fig. 4). The model showed high robustness with values of R^2^ × (0.99; 0.95), R^2^Y (0.99;0.98), and Q2 (0.97; 0.93) at 24 and 48 h, respectively. Compact points are visualized by clusters in each treatment. The statistical validity of the model was confirmed by a cross-validation analysis of variance, which demonstrated robust significance (*p* < 0.03), ruling out the risk of overfitting.

**Fig. 5:**
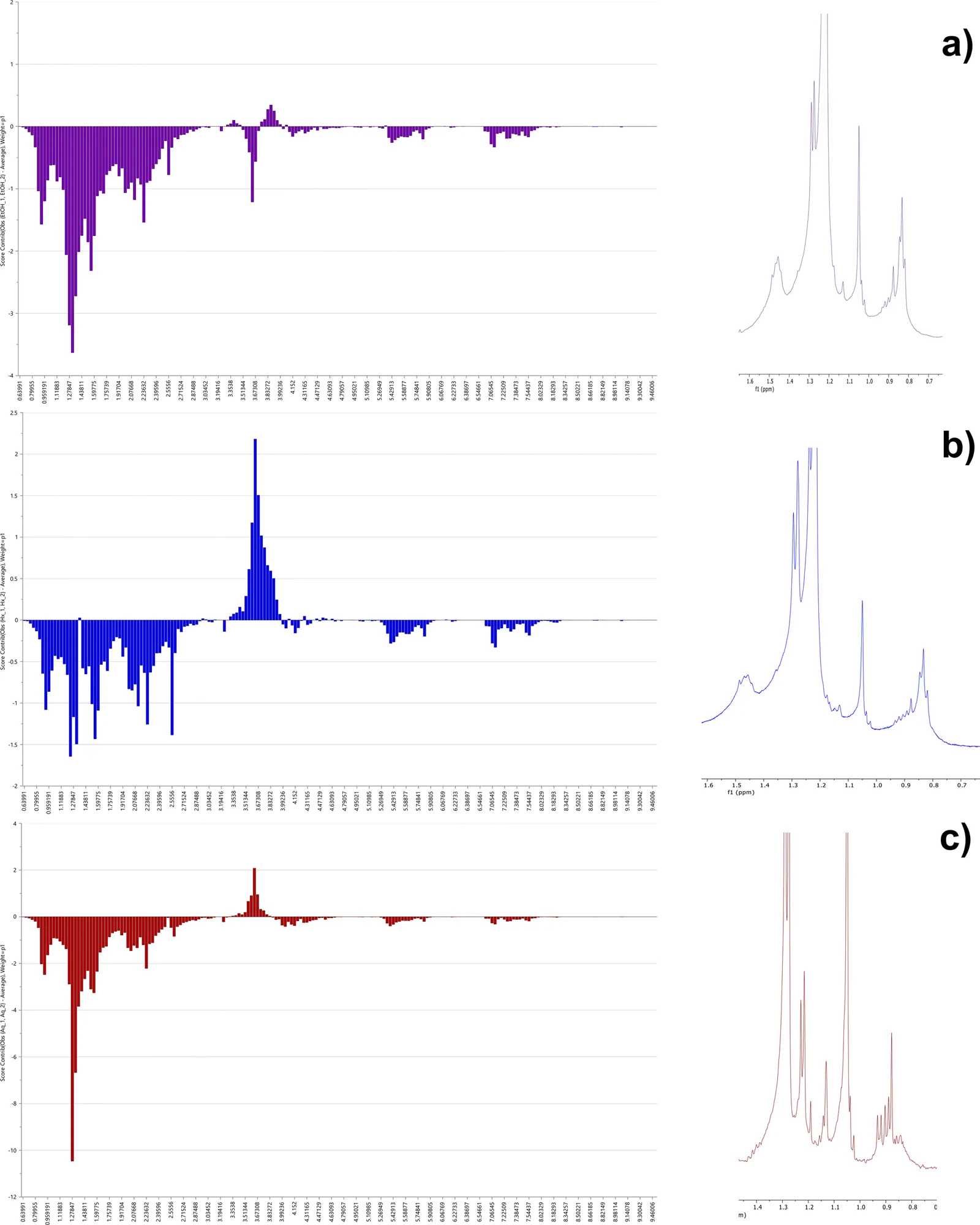
The contribution plots illustrate the variable contributions to discriminate each treatment *vs.* the average, 5a) EtOH: H_2_O, 5b) FR_Hx, and 5c) FR_Aq, at 48 h. Positive bars with higher intensity contribution indicate a high scratched area percentage, while negative bars with high intensity contribution indicate a decrease in scratched area percentage

Contribution plots were generated to identify the main variables contributing to the reduction in the scratched area, comparing the most active treatments with the average across all treatments at 24 h (Online Resource 1) and 48 h (Fig. 5).

The EtOH: H_2_O extract (Fig. 5a) shows that the signals in the peaks 1.11 to 1.8 ppm have the highest contributions, indicating that their above-average negative intensities are responsible for the decrease in the scratched area, suggesting that their decreased intensity could be attributed to the presence of alkanes. Comparing the FR_Hx extract (Fig. 5b) showed the strongest signals responsible for the scratch area decrement: negatively at *δ* 0.8 to 2.23 ppm. Finally, FR_Aq (Fig. 5c) suggests a negative intensity with a decrease effect in scratched area at *δ* 1.1 to 1.5 ppm. Consistently, in FR_Hx and FR_Aq spectras, from *δ* 3.31 to 3.9 ppm the contribution is positive with high intensity attributed to a high scratched area percentage. The region at *δ* 0–2 ppm is represented in the extract and fractions with a methyl group in fucoidan, furthermore, alcohol proton signals including mannitol, at *δ* 3–4 ppm negatively slightly regulated, could be part of the effect on scratched area closing response.

### The phagocytic activity of macrophages by the effect of *E. menziesii* extract and its fractions

**Fig. 6:**
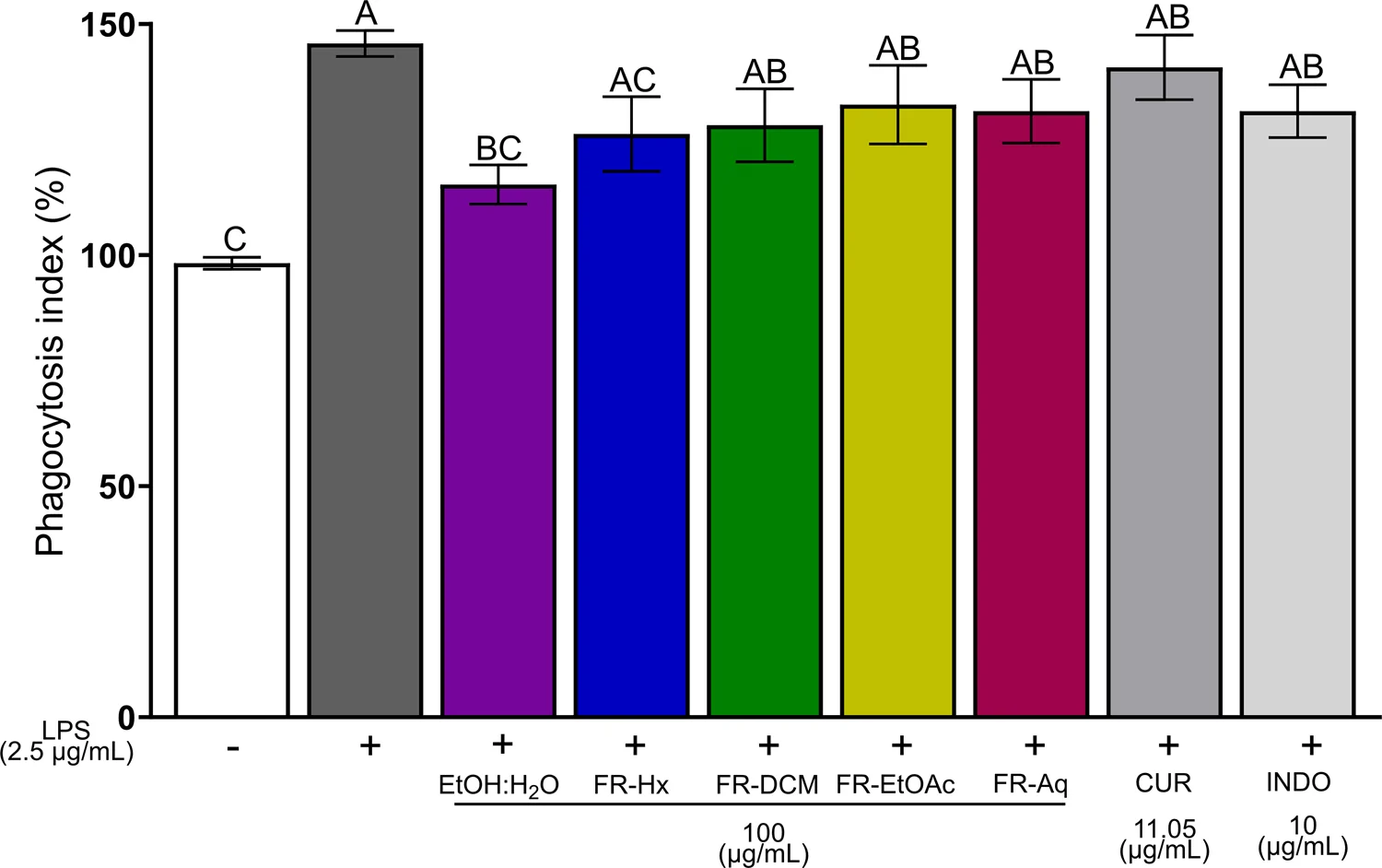
Relative Phagocytosis Activity of macrophages treated with *E. menziesii*’s extracts. Data are presented as the mean ± SEM per duplicate (24 h). A two-way ANOVA was performed to evaluate differences across groups, followed by Tukey’s post hoc test for multiple comparisons. Nomenclature a-c: The use of identical lowercase letters within each bar indicates no significant difference between treatment values; conversely, differing letters denote statistically significant differences within treatments (*p* < 0.05)

Macrophages, well known as antigen-presenting cells (APC), play a role in the sensing of antigen presence and as waste collectors of cells through phagocytosis (Fig. 6). Relative phagocytic activity was increased in the LPS+ group compared to the LPS– group (*p* < 0.0001). EtOH: H_2_O extract was lower than in the LPS+ control group (*p* < 0.03). The FR_DCM, FR_EtOAc, and FR_Aq fractions increased the phagocytic activity compared to the LPS– control group (*p* < 0.04, 0.007, and 0.01, respectively). The FR_Hx had no significant differences compared to the control groups. The CUR and INDO groups showed significantly increased activity compared with the LPS– control group (*p* < 0.0002 and 0.01, respectively).

### Pro-inflammatory cytokine expression and protein levels regulated by *E. menziesii*

**Fig. 7:**
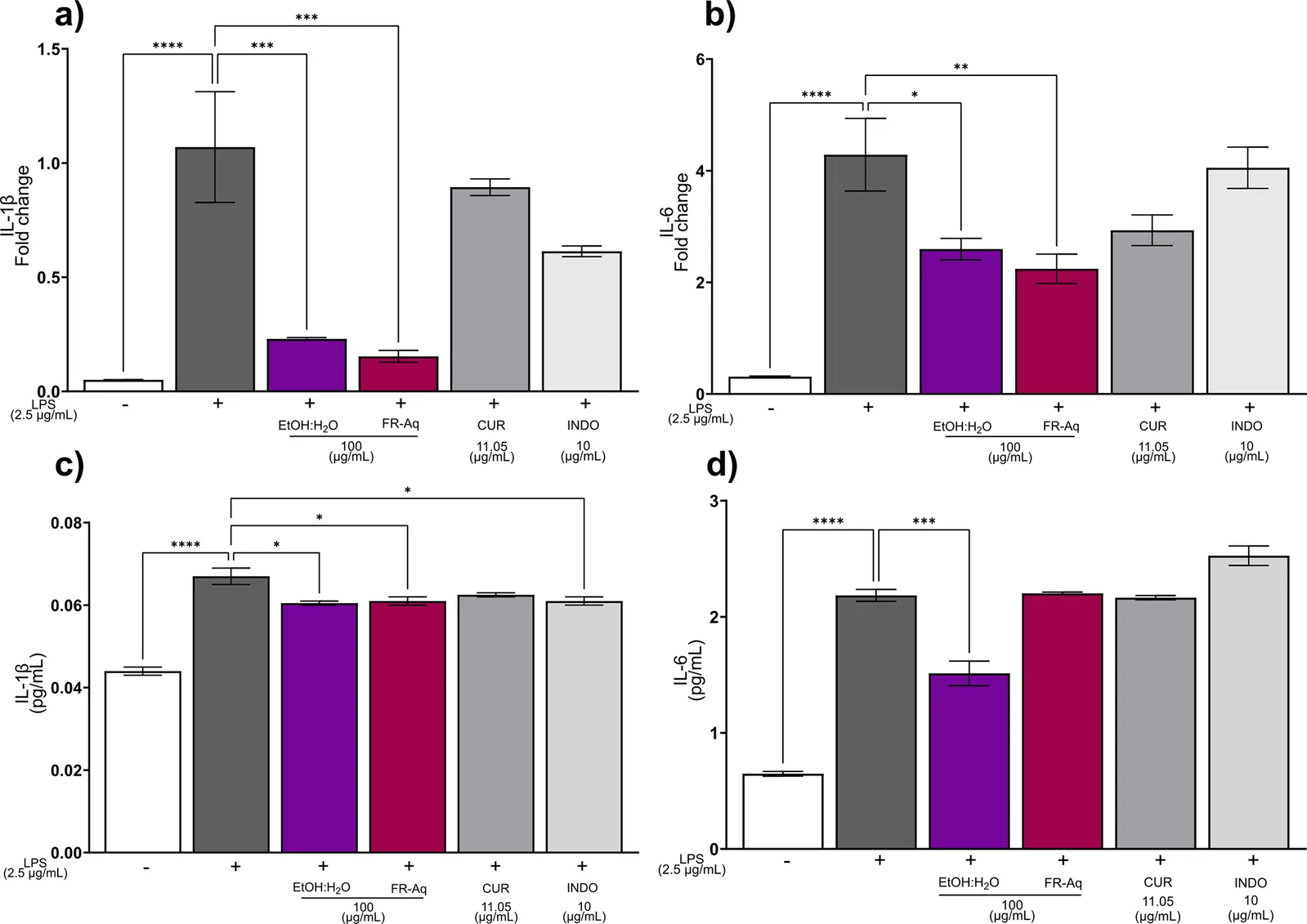
Proinflammatory IL-1β and 6 cytokines mRNA and protein levels in RAW 264.7 macrophages treated with *E. menziesii*’s extracts. (a) IL-1β fold change (*p* < 0.0001**** and 0.0003***) and (b) IL-6 fold change (*p* < 0.0001****, 0.006* and *p* < 0.2*) in mRNA levels. (c) IL-1β (*p* < 0.0001**** and *p* < 0.03*) and (d) IL-6 protein levels (*p* < 0.0001**** and *p* < 0.008***). Data are presented as mean ± SEM from triplicate measurements (24 h). A one-way ANOVA was performed to assess differences between groups, with Dunnett’s test vs. LPS + control group

The mRNA and protein levels of the proinflammatory cytokines IL-1β and IL-6 were analyzed as a consequence of macrophage M1 polarization induced by LPS (Fig. 7). Compared to the LPS+ control group, the EtOH: H_2_O extract significantly reduced IL-1β expression (*p* < 0.0003) and showed a tendency to reduce IL-6 expression (*p* < 0.02). The FR-Aq demonstrates significant reductions in both IL-1β and IL-6 levels (*p* < 0.0001 and *p* < 0.06, respectively). Moreover, the CUR and INDO groups did not exhibit a significant reduction in cytokine expression compared to the LPS+ control group.

Furthermore, IL-1β protein levels were significantly lower than in the LPS+ control group (*p* < 0.0001). The EtOH: H_2_O and the FR-Aq treatments reduced interleukin levels, similarly to the effects observed with INDO treatment (*p* < 0.03). Finally, IL-6 levels increased notably in the control group LPS+ (*p* < 0.0001), whereas only the EtOH: H_2_O extract significantly decreased IL-6 levels (*p* < 0.0008).

## Discussion

Inflammation remains a prominent health concern. Since NSAIDs and corticosteroids (primary prescription medications employed in the management of acute and chronic inflammation) may induce gastrointestinal complications, ulcers, and elevate cardiovascular risks such as heart attacks or strokes with prolonged use, the investigation of novel potential compounds remains a vital area of research [26].

Although there are studies on the search for compounds in brown seaweed and most of the research of *E. menziesii* brown seaweed is related to its environmental impact and morphological characteristics, no research has been conducted on the effects of *E. menziesii* extracts on pro-inflammatory mechanisms following LPS-induced macrophage activation, which would allow for a broader study of marine organisms with biological potential. This study aimed to assess the effect of *E. menziesii* EtOH: H_2_O extract and its fractions on the core elements of the immune system. The first step was to characterize the EtOH: H_2_O and fractionated extracts of *E. menziesii* by ^1^H-NMR spectroscopy, following the methodology outlined for the characterization of *Saccorhiza polyschides* extracts [27]. It was found that the protons in the region 0.91–2.86 ppm could be attributed to lipophilic compounds such as fatty acids, sterols, terpenes, and other lipids [28]. The FR-Aq shows strong signals between 3 and 4 ppm, likely attributable to mannitol, a widely recognized polyol found in various brown seaweeds [29]. ATR/FT-IR spectroscopy was previously used to analyze *E. menziesii*, revealing the asymmetric bending vibrations (1404–1417 cm cm^− 1^) of the CH₃ and -OH groups, along with the symmetric stretching vibrations of the sulfate and C-O-S groups (824.84–893.38 cm cm^− 1^) [17]. Hence, spectroscopic elucidation of the compounds indicates that *E. menziesii* extracts are rich in sulfated polysaccharides and mannitol.

It is important to note that in traditional medicine, whole plants or plant combinations were used rather than individual compounds. The application of seaweed as a source of bioactive extracts obtained as crude extracts has generated increasing interest in recent years, especially for nutraceutical and biomedical uses. Evidence suggests that crude ethanol: water extracts frequently exhibit greater bioactivity *in vitro* and/or *in vivo* than isolated components at equivalent doses [10, 30].

The sulfated polysaccharides derived from various species of algae have been demonstrated to possess anti-inflammatory properties, including the reduction of IL-1β and IL-6 production [31]. This anti-inflammatory effect might be attributed to enhanced sulfation, which increases the presence of negatively charged molecules that interact with proinflammatory agents. The structure, dimensions, and overall charge of polysaccharides significantly influence their attraction to inflammatory mediators, as larger molecules may possess more extensive binding sites, while charge distribution influences electrostatic interactions [32]. Specifically, fucoidan, a sulfated polysaccharide present in the cell walls of seaweeds (primarily brown), exhibits anti-inflammatory activity. The chemical composition of fucoidan is generally highly complex and varies substantially depending on the algal source, geographic location, and extraction technique. Nevertheless, its structure comprises recurring l-fucopyranose units connected via α-(1→3) bonds or alternating l-fucopyranose residues linked through α-(1→3) and α-(1→4) bonds [33]. Several studies support the idea that its anti-inflammatory activity is mediated by interaction with COX-2, thereby blocking the conversion of arachidonic acid to prostaglandin E2, which reduces various inflammatory parameters [34]. Furthermore, fucoidan, derived from *Fucus vesiculosus*, reduced the release of TNF-α and IL-1β in RAW 264.7 macrophages and restricted neutrophil infiltration, indicating its ability to prevent the initial phases of inflammation [35]. Comparable findings have been reported for fucoidan obtained from *Ecklonia maxima*, as the fucoidans significantly reduced the production of NO and the levels of TNF-α, IL-1β, and IL-6 in LPS-stimulated RAW 264.7 cells [36]. Moreover, NFκB acts as a crucial transcription factor in immune activation, and reductions in p65 NFκB subunit and evasion of IκB phosphorylation are associated with fucoidan treatment [37]. Consequently, NFκB regulates pro-inflammatory cytokines, reducing their levels in various cells and tissues.

On the other hand, the chemical analysis of *E. menziesii* extracts also identified signals characteristic of the sulfated polysaccharide fucoidan and the sugar alcohol mannitol, in addition, the chemometric analysis showed that the effect can be attributed to these compounds, which are naturally found in brown seaweeds and forms part of their metabolism. Mannitol functions as a carbon source, facilitates translocation, and acts as an osmolyte solvent [38]. Literature indicates its potential application in surgical procedures to reduce intracranial pressure in cases of neuroinflammation mediated by microglial activity. An elevation in plasma osmolality may influence immune cell functions, including degranulation, adhesion, and phagocytic capacity, among others [39]. The increased mannitol content in *Sargassum ilicifolium* significantly promotes wound healing and modulates inflammatory responses [29]. Thereby, its abundant presence in the extracts provides evidence of promising anti-inflammatory activity.

Macrophages are crucial for antigen clearance of antigens and in the activation of more specialized immune cells through the secretion of cytokines, chemokines, and mediators that modulate vascular responses. Accordingly, the RAW 264.7 cell line was used to evaluate the anti-inflammatory potential of *E. menziesii*, as it has been demonstrated to be an effective *in vitro* model of inflammation [20]. However, our research involved evaluating the biosafety of the *E. menziesii* extract in RAW 264.7 macrophages. The effect of *E. menziesii* extracts on cell viability has been described in other cell types and complete organism models. Previously, our research group demonstrated that the methanolic extract did not decrease the viability of brine shrimp *Artemia salina*, nor the nervous system cell cultures, nor of human peripheral blood lymphocytes [18]. Using *E. menziesii* extract and its fractions, similar favorable results have been observed, as the concentrations used in the present study did not significantly reduce RAW 264.7 cell viability, with IC_50_ values exceeding 1000 µg/mL. These findings are comparable to those obtained from various extracts of brown seaweeds. For example, extracts of *Sargassum horneri*, *Sargassum fusiforme*, and *Undaria pinnatifida* did not induce cytotoxic effects at concentrations exceeding 400 µg/mL [40]. Further, treatment with *Sargassum coreanum* extract did not significantly reduce cell viability at concentrations of 25, 50, 100, and 200 µg/mL in RAW 264.7 macrophages [41]. Crude polysaccharides and fucoidan fractions derived from *Sargassum autumnale* were evaluated at concentrations of 25, 50, 100, 200, and 400 µg/mL; only at 400 µg/mL cell viability was decreased, leading to its exclusion from subsequent experiments [42].

Following the chemical characterization and safety evaluation in RAW 264.7 macrophages, *in vitro* cell scratch assays are considered straightforward and relatively cost-effective methods for assessing directional cell migration [43]. The evaluation period for the scratch assay was conducted at 24 and 48 h, in line with the MTT viability assay. The latter functioned as a parallel control to observe increases in cell viability, confirming that at 48 h, cell viability was maintained to 100%. Therefore, the closure of the scratch can be attributed to macrophage migration. LPS, the predominant component of Gram-negative bacterial cell walls, serves as the classic inducer of the inflammatory cascade. Beyond its bioactivity, toxicity, and immunomodulatory effects, which influence wound healing and scar formation, the inflammatory response elicited by LPS has significant implications for wound repair, reflected in scratch assays [44]. Fibroblasts and macrophages are essential components in the process of wound healing. Circulating and tissue-resident macrophages are essential, as they eliminate potential pathogens and, once the pathogens are cleared, reduce inflammation, which is critical for initiating and sustaining tissue remodeling and regeneration [29]. During the initial acute inflammatory phase of wound healing, neutrophils and M1-like monocytes/macrophages are the primary cellular constituents of the skin wound microenvironment. In therapeutic applications, *E. menziesii* counteracts the effects of LPS by promoting scratched area closure, with M1 macrophages arriving at 24 and 48 h in the EtOH: H_2_O extract and FR-Aq treatments. Polarized migrating macrophages are characterized by their phagocytic activity, which encompasses the ability to recognize, eliminate, and present antigens to other lymphoid cells. Research indicates that compounds contained in extracts of the algae *Sargassum horneri*, *Sargassum fusiforme*, and *Undaria pinnatifida* serve as mediators of enhanced phagocytic activity, particularly at concentrations below 200 µg/mL [40]. *Hizikia fusiforme* is another algae species that promotes phagocytic activity [45]. Additionally, the brown algae *Sargassum thunbergii* exhibits increased cellular phagocytosis attributable to its high levels of fucose and sulfated polysaccharides [46]. Taken together, these findings reveal that some seaweed compounds are notably associated with increased phagocytic activity through the upregulation of TLR-4 expression [47].

The RAW 264.7 mouse peritoneal macrophage cell line is classically used for *in vitro* screening of anti-inflammatory parameters. Stimulated cells produce inflammatory substances such as nitric oxide (NO), derivatives of arachidonic acid, and pro-inflammatory cytokines, which activate signaling pathways including NF-κB and MAPK [42]. The present study demonstrated that LPS treatment increased the release of proinflammatory cytokines in RAW 264.7 cells. Co-treatment with *E. menziesii* extract and the FR-Aq fraction significantly reduced the expression of IL-1β and IL-6, whereas only the EtOH: H_2_O extract showed effects at both the protein and mRNA levels. Additionally, other brown seaweeds exhibited decreased levels of pro-inflammatory parameters. *Dictyopteris divaricata*, *Dictyopteris prolifera*, *Prionitis cornea*, *Grateloupia lanceolata*, and *Grateloupia filicina* significantly decreased TNF-α and IL-6 production [48]. The *Undaria pinnatifida* treatment reduces pro-inflammatory cytokines (TNF-α, IL-1β, IL-6), decreases ROS production, and suppresses iNOS and COX-2 activity, thereby mitigating oxidative and inflammatory damage *in vitro* [49]. Finally, the identified compounds exhibit anti-inflammatory effects, as previously described. Fucoidan derived from *Sargassum fusiforme* demonstrates its proinflammatory IL- 1β, IL-6, and TNF-α decrement by the NFκB regulation [37]. Sulfated polysaccharides obtained from *Sargassum fulvellum* reduce the production of proinflammatory cytokines at concentrations below 100 µg/mL [31]. Phlorotannins isolated from *Ecklonia cava* decreased NFκB and p50 p65 subunits with inhibition in binding to TNF-α and IL-6 promoters [50]. Furthermore, mannitol reduced markers of the microglia/macrophage M1-like phenotype (nuclear p65, TNF, and iNOS), decrementing microglial activation *in vivo *[39].

## Conclusion

This study demonstrated the potential of the extract and fractions of *E. menziesii* with the presence of compounds associated with a decrease in proinflammatory parameters, through the characterization of the algal extracts and fractions by ^1^H-NMR and their comparison with prior studies. As a precautionary measure, cell viability analysis confirmed that the algal concentrations do not cause cytotoxicity on this cell line. Furthermore, their effect on scratched closure area reveals that the FR_Aq has a similar effect to that of the EtOH: H_2_O extract, reversing the LPS effect. In a complementary manner, chemometric analysis demonstrated that signals associated with mannitol and sulfated polysaccharides could be responsible for the reduction in scratched closure area. The phagocytic activity suggests that the extracts and fractions obtained from *E. menziesii* do not contain compounds that trigger this process more significantly than the LPS+ group. Furthermore, only the EtOH: H_2_O extract reduced levels to baseline lysosomal activity. The observed trend indicates a decrease across the fractions. Additionally, the findings demonstrate that both extracts reduce the expression of IL-6 and IL-1β. Nevertheless, while the effect of the EtOH: H_2_O extract is evident at the protein level, the FR_Aq fraction suggest that further research and the addressing of existing limitations are required.

However this research presents certain limitations. Initially, although the extract and fractions has been chemically characterized and metabolomic analysis may facilitate the identification of the most active compounds, it is important to acknowledge that these compounds may exhibit synergistic interactions or potentially antagonize each other. Secondly, since a murine cell line was used, the *in vitro* cellular environment remains over simplified, based on a single cell type cultivated in a monolayer or suspension. Nevertheless, the data generated by such cellular systems aim to accurately represent the complex interactions among different cell types and extracellular matrices within an *in vivo* environment. Furthermore, the use of cell culture reduces the necessity for animal sacrifices during toxicity assessments of various compounds; however, it is unable to account for metabolic responses or reactions, or to monitor the diverse behavioral responses to the treatment. Although the threshold was used in the analysis of the scratched area to exclude cells located within that area, and the MTT assay ruled out an increase in proliferation, given the wide variety of wound-induction techniques available and the numerous experimental design factors that can influence the results, it remains difficult to make a direct comparison between the different studies. RAW 264.7 macrophages are considered semi-adherent cells. During the adhesion process, certain cells may remain loosely attached, allowing some cells to remain in suspension, particularly if the culture area has reached confluence. This mechanism facilitates the repositioning of some cells within the seeding area. Further studies should analyze the impact of the extract and its fractions on human cell lines and in vivo models. Finally, inflammation is a complex process involving numerous coordinated variables. This study explores the effect on macrophage cells and two proinflammatory cytokines, with their possible effect reversing the LPS interaction. It is necessary to explore other mechanisms associated with inflammation (NO quantification, iNOS protein levels, anti-inflammatory cytokine measurement, and metabolomic studies).

## Supplementary Information

Below is the link to the electronic supplementary material.

Supplementary Material 1

Supplementary Material 2

## Data Availability

The datasets generated during the current study are available in the Figeshare repository, https://doi.org/10.6084/m9.figshare.32494050.

## Abbreviations

LPS: Lipopolysaccharides
^1^H-NMR: Proton Nuclear Magnetic Resonance
MVDA: Multivariate Data Analysis
IL-1β: Interleukin-1β
IL-6: Interleukin-6
NSAIDs: Non-steroidal anti-inflammatory drugs
OPLS: Orthogonal Partial Least-Squares
MTT: Thiazolyl Blue Tetrazolium Bromide
DMSO-d6: Dimethyl sulfoxide-d6
TMS: Tetramethylsilane
4-HBA: 4-Hydroxybenzoic acid
HMDB: Human Metabolome Database
RPMI: Roswell Park Memorial Institute
FBS: Fetal Bovine Serum
H_2_O_2_: Hydrogen peroxide
PCR: Polymerase Chain Reaction
PBS: Phosphate Buffered Saline
SEM: Standard Error of the Mean
E_t_OH : H_2_O: Ethanol: water (1:1 v/v)
FR_Hx: Hexane
FR_DCM: Dichloromethane
FR_E_t_OA_c_: Ethyl Acetate
FR_Aq: Aqueous
APC: Antigen-presenting cells
