# Supplementary material for: Chemical profiling of Egregia menziesii and anti-inflammatory potential in LPS-activated RAW 264.7 macrophages
Source: Mol Biol Rep. 2026 Sep 5;53(1):1529. doi: 10.1007/s11033-026-12685-y (PMC13546325; doi:10.1007/s11033-026-12685-y)
Supplement: Supplementary file 1 [file 11033_2026_12685_MOESM1_ESM.pdf]

Table 1. Primer sequences for gene amplification.

cDNA	Sequence
IL-1β	F-5′-GCACTACAGGCTCCGAGATGAA-3′
	R-5′- GTCGTTGCTTGGTTCTCCTTGT-3′
IL-6	F-5′-TACCACTTCACAAGTCGGAGGC-3′
	R-5′- CTGCAAGTGCATCATCGTTGTTC-3′
GAPDH	F-5′-CAAGGTCATCCATGACAACTTTG-3′
	R-5′- GGGCCATCCACAGTCTTCTG-3′

Table 2. Yield of extracts and their fractions from Egregia menziesii.

**Extract of Fraction (Abbreviation)**	**Grams (g)**	**Yield (%)**
Ethanol:water 1:1 (EtOH:H_2O)	6.248	24.82
Hexane (FR-Hx)	1.186	18.98
Dichloromethane (FR-DCM)	0.067	1.07
Ethyl Acetate (FR-EtOAc)	0.066	1.05
Aqueous (FR-Aq)	4.247	67.97

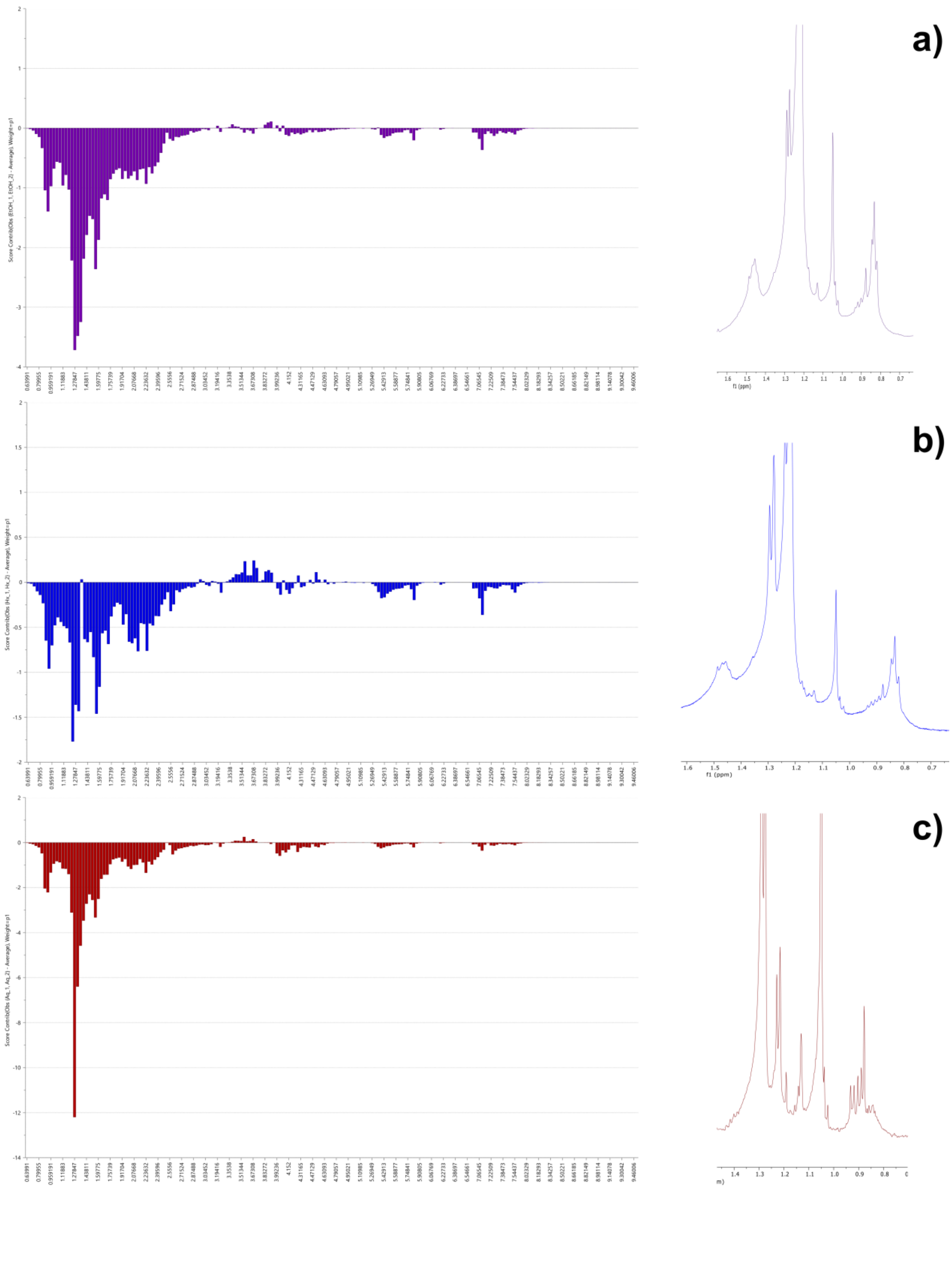

Fig. 1 *The contribution plots illustrate the variable contributions to discriminate each treatment vs the average (1a, 1b, and 1c), at 24 h. Positive bars with higher intensity contribution indicate a high wound area percentage, while negative bars with high intensity contribution indicate a decrease in wound area percentage*
